# Supplementary material for: Causal Relationship between Aging and Anorexia Nervosa: A White-Matter-Microstructure-Mediated Mendelian Randomization Analysis
Source: Biomedicines. 2024 Aug 16;12(8):1874. doi: 10.3390/biomedicines12081874 (PMC11351342; doi:10.3390/biomedicines12081874)
Supplement: Supplementary file 1 [file biomedicines-12-01874-s001.zip › Supplementary Figure/Figure S4/Singe SNP plot of MR analysis for TL to ubm-b-2107.pdf]

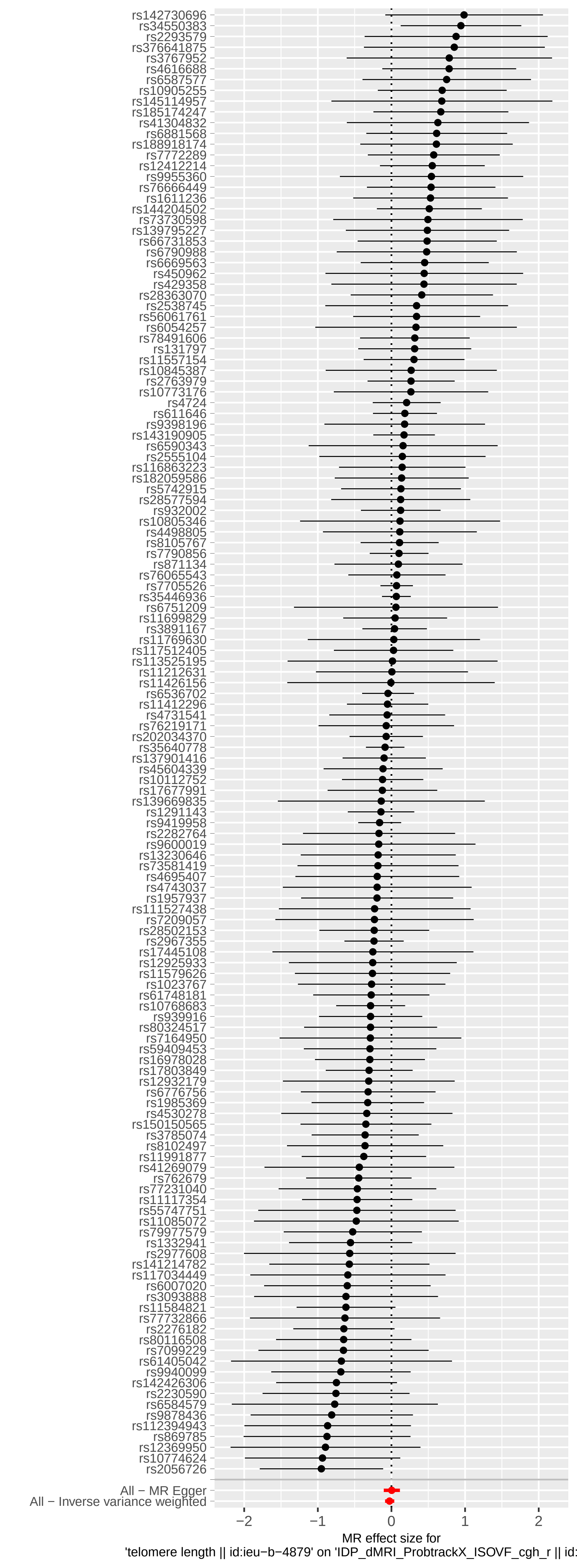

rs142730696
rs34550383
rs2293579
rs376641875
rs3767952
rs4616688
rs6587577
rs10905255
rs145114957
rs185174247
rs41304832
rs6881568
rs188918174
rs7772289
rs12412214
rs9955360
rs76666449
rs1611236
rs144204502
rs73730598
rs139795227
rs66731853
rs6790988
rs6669563
rs450962
rs429358
rs28363070
rs2538745
rs56061761
rs6054257
rs78491606
rs131797
rs11557154
rs10845387
rs2763979
rs10773176
rs4724
rs611646
rs9398196
rs143190905
rs6590343
rs2555104
rs116863223
rs182059586
rs5742915
rs28577594
rs932002
rs10805346
rs4498805
rs8105767
rs7790856
rs871134
rs76065543
rs7705526
rs35446936
rs6751209
rs11699829
rs3891167
rs11769630
rs117512405
rs113525195
rs11212631
rs11426156
rs6536702
rs11412296
rs4731541
rs76219171
rs202034370
rs35640778
rs137901416
rs45604339
rs10112752
rs17677991
rs139669835
rs1291143
rs9419958
rs2282764
rs9600019
rs13230646
rs73581419
rs4695407
rs4743037
rs1957937
rs111527438
rs7209057
rs28502153
rs2967355
rs17445108
rs12925933
rs11579626
rs1023767
rs61748181
rs10768683
rs939916
rs80324517
rs7164950
rs59409453
rs16978028
rs17803849
rs12932179
rs6776756
rs1985369
rs4530278
rs150150565
rs3785074
rs8102497
rs11991877
rs41269079
rs762679
rs77231040
rs11117354
rs55747751
rs11085072
rs79977579
rs1332941
rs2977608
rs141214782
rs117034449
rs6007020
rs3093888
rs11584821
rs77732866
rs2276182
rs80116508
rs7099229
rs61405042
rs9940099
rs142426306
rs2230590
rs6584579
rs9878436
rs112394943
rs869785
rs12369950
rs10774624
rs2056726
All − MR Egger
All − Inverse variance weighted
−2
−1
0
1
2
MR effect size for
'telomere length || id:ieu−b−4879' on 'IDP_dMRI_ProbtrackX_ISOVF_cgh_r || id:
